# Leg Press vs. Smith Machine: Quadriceps Activation and Overall Perceived Effort Profiles

**DOI:** 10.3389/fphys.2018.01481

**Published:** 2018-10-23

**Authors:** Gian Mario Migliaccio, Antonio Dello Iacono, Luca Paolo Ardigò, Pierre Samozino, Enzo Iuliano, Zoran Grgantov, Johnny Padulo

**Affiliations:** ^1^Sport Science Lab, Cagliari, Italy; ^2^Wingate Institute, The Academic College at Wingate, Netanya, Israel; ^3^Institute of Clinical Exercise and Health Science, School of Health and Life Sciences, University of the West of Scotland, Hamilton, United Kingdom; ^4^Department of Neurosciences, School of Exercise and Sport Science, Biomedicine and Movement Sciences, University of Verona, Verona, Italy; ^5^Laboratoire Interuniversitaire de Biologie de la Motricité, EA 7424, Université Savoie Mont Blanc, Chambéry, France; ^6^Faculty of Psychology, eCampus University, Novedrate, Italy; ^7^Faculty of Kinesiology, University of Split, Split, Croatia

**Keywords:** muscle strength, strength exercise, workload, resistance training, exercise intensity

## Abstract

First aim was describing Smith machine squat and leg press exercise as nominal load, knee extensors activity, and rating of perceived exertion. Second aim was developing predictive equations to provide same muscular activation and same perceived exertion nominal loads during the two exercises. To do that, *vastus lateralis* and *vastus medialis* activation, as their summed surface electromyography signal integrals, and overall perceived exertions were measured at different nominal loads during Smith machine squat and leg press exercise in adult male athletes experienced in weight training. Correlation and multistep stepwise analyses were performed. Then, two different results-driven predictive equations to provide same electromyography signals and same perceived exertion nominal loads were developed. The same electromyography signal equation results were less accurate (i.e., less predictive) due to high inter-individual differences, whereas the same perceived exertion equation results were more accurate, because perceived exertion is more related to the Smith machine squat and leg press exercise overall level of exertion than to the two single muscles that were investigated. In conclusion, these two equations represented an initial attempt to provide athletes and coaches with a new tool to mutually convert equivalent nominal loads during Smith machine squat and leg press exercise over a training period.

## Introduction

Closed kinetic-chain exercises (CKC, i.e., exercises performed where hand or foot is fixed in space and cannot move Blackburn and Morrissey, 1998, e.g., a leg press exercise [LP]) are widely used in rehabilitation settings and as a training means within neuromuscular training programs to promote functional movement performance. These resistance exercises are commonly prescribed as primary training modalities, aimed at strengthening lower limb muscles and developing functional enhancements based on their biomechanical similarities to many typical athletic movements (Escamilla et al., 1998; Hopkins et al., 1999). CKCs involve muscles' concurrent contraction to improve their dynamic stability to refine proprioception, as well. The opposite of CKCs are open kinetic chain exercises (OKC, i.e., exercises performed where hand or foot is free to move1). According to literature, completely or partially—e.g., bar-guided—OKC squat (e.g., a Smith machine squat, for instance a squat exercise performed at the Smith machine Multipower [MP], read below) is generally preferred over CKC machines (e.g., leg press) by strength-training athletes, because the former exercise is thought to provide a more unstable exercise requiring a greater recruitment of trunk and lower limb musculature (Anderson and Behm, 2005; Schwanbeck et al., 2009). In fact, several studies indicate that OKCs may be preferable over CKC machines for recruiting the major muscle groups of the legs (Hogan, 1984; Haff, 2000; Cotterman et al., 2005). Closed kinetic-chain exercise machines, however, are easier to use by beginners and those with injuries in the early to mid-stages of rehabilitation protocols, as they are easier to control, require less trainer supervision compared to OKCs and expose the exerciser to lower overall injury risk (Haff, 2000). Accordingly, LP is commonly indicated for rehabilitation of the knee, especially in the rehabilitation of athletes who cannot tolerate full weight-bearing during squatting movements (Hasselgren et al., 2011). Muscles' electromyography activity (EMG; Kamen, 2014), a proxy for physical activity (i.e., mechanical output), and rating of perceived exertion (RPE; Borg, 1982), a proxy for metabolic expenditure (i.e., metabolic input), are acknowledged different effort outcomes. In addition, it is acknowledged that both of them increase similarly over increasing mechanical external load (Lagally et al., 2002, 2004).

Although the specific configuration of the above different exercises' variations can reasonably imply a selective muscle activity pattern with specific mechanical outcomes such as force or torque, power, and muscle activity, no studies have attempted to compare the acute responses associated to these two common training modalities, i.e., LP and MP. To date, a paucity of research (Escamilla et al., 2001) has investigated these two exercises, which are characterized by a similar movement pattern. However, individual differences can significantly influence muscle activity pattern. These individual differences are related to anthropometric characteristics, level of training, physical activity backgrounds, etc. LP and MP are two multijoint exercises with a high number of muscles involved during movement. So, it is not possible to have a standard muscle activity pattern also when kinematic is standardized. Within this context, the ability to choose specific load configurations for effective training processes, or—more interestingly—the ability to determine an easy-to-use method able to provide a practical mutual conversion equation between the two training modes' nominal loads, may be very useful for everyday practice. Variables candidates for being assessed as *criteria* ones to develop such a method could be muscle activity and/or perceived exertion. In other words, it could be investigated an eventual correspondence between same muscle activity and/or perceived exertion at same effective load (but different nominal load) with different training modes (Stensdotter et al., 2003). Athletes or their trainers could be interested in switching the two training modes each other while keeping the same effort, e.g., from LP to MP, over improvement from beginner to trained state, from post-injury to almost-recovered condition, or even to challenge any eventual training aversion issue.

In this regard, the traditional determination of the specific workload at which mechanical outcomes (e.g., force or torque, and muscle activity) are maximized is derived from the calculation of the measured or predicted one-repetition *maximum* (Pereira and Gomes, 2003; Nuzzo et al., 2008; Hoffman et al., 2009). Otherwise, as a valid and reliable alternative, workload determination relies on the assessment of the power-force-velocity profiling for more individually-addressed protocols (Loturco et al., 2015; Morin and Samozino, 2016). As a consequence, such assessment methods are operated on a regular basis, since they are based on common and sport-specific movements and can therefore be used for long-term monitoring and training processes (Cross et al., 2017). The potential findings of such a comprehensive evaluation and comparison between a MP and a LP may provide practical guidelines for suggesting combined, alternate, or progressive applications of these two exercises into a training design aimed at achieving specific goals.

Therefore, the aims of the current study were (1) to assess/compare knee extensor muscles' EMG during MP and LP over a gradually increasing load; and (2) to investigate relationships with accompanying predictive equations describing the mutual load conversion between the two exercises, taking into account load, EMG, and RPE.

## Materials and Methods

### Participants

Sixteen male athletes (age 27.7 ± 7.3 [mean ± SD] yrs, height 1.79 ± 0.07 m, mass 75.6 ± 13.5 kg, body mass index 23.5 ± 2.3 kg·m^−2^), and 11.07 ± 2.69 years of experience in weight training with a weekly training frequency of 2.40 ± 0.63 sessions/week participated in the study. In order to ensure safety of procedures and to avoid bias on results due to an incorrect execution of the exercises, following inclusion *criteria* were used: at least 5-year experience in powerlifting or weight training and absence of musculoskeletal injury at the time of participation in the study or in the previous 12 months. Following exclusion *criteria* were instead applied: use in the previous 2 months of drugs or other substances potentially influencing subjects performance, use in the previous 24 h of caffeine, alcohol, or other energetic drinks, and abstention from intense exercise in the previous 24 h. This study was carried out in accordance with the recommendations of the Code of Ethics of the World Medical Association, University of Split Ethics Committee. The protocol was approved by the University of Split Ethics Committee. All subjects gave written informed consent in accordance with the Declaration of Helsinki.

### Protocol

The participants took part in the experimental protocols on different days at the same time of the day (2.00 until 4.00 p.m.) to eliminate any influence of circadian variation (Ammar et al., 2015), with three recovery days in-between, and under controlled environmental conditions (23.2 ± 0.5°C temperature and 55.1 ± 1.8% relative humidity). The first day was dedicated to familiarization with the MP (with Multipower, Line Selection, Technogym, Gambettola, Italy) and LP (with Leg press, Line Selection, Technogym, Gambettola, Italy), performed in randomized order (i.e., participants were asked to freely exercise—under operators supervision—with the two machines for about half an hour). The second day was dedicated to assessing EMG data during both the MP and LP in randomized order.

**Figure 1:**
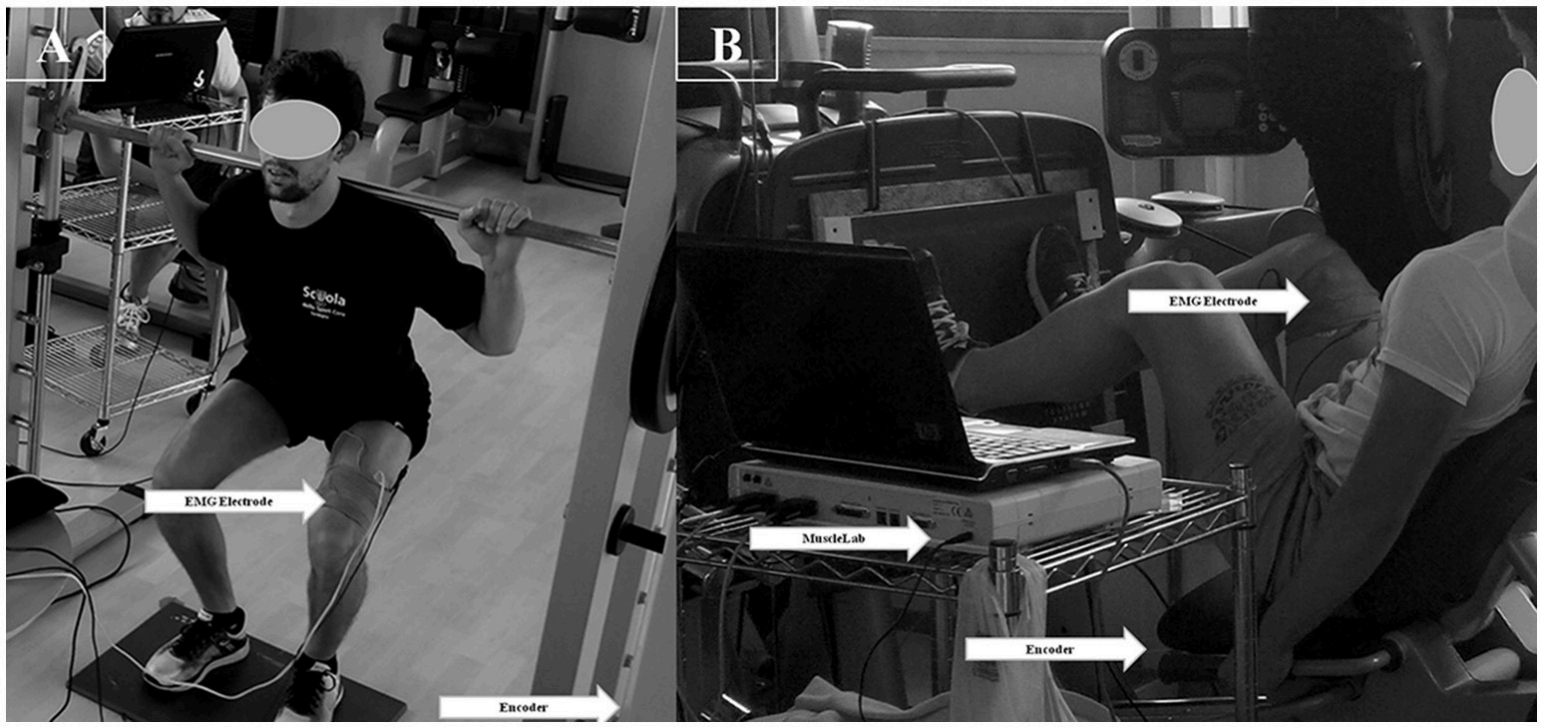
Participant exercising at Multipower **(A)** and at Leg press **(B)**. EMG electromyography activity. Written informed consent for the publication of image was obtained from participant.

During the second day, the participants started with a standardized warm-up routine (Padulo et al., 2015a) consisting of five repetitions (with 30-s recovery in-between) pushing 30% of their body weight at a self-selected low speed during both the MP (with the same 17-kg barbell, Figure 1A) and LP (with the same 49-kg seat, Figure 1B). Feet were always placed parallel each other and at shoulder-width. It must be noticed that LP effective seat weight results from the multiplication of seat's 49-kg mass by the sine of 10.2°, which was the inclination of the sliding axis of the LP seat over the horizontal (Padulo et al., 2017). Both machines (for the MP and LP) were checked before and after the experiments (Winter, 2012) with calibration loads (5, 10, 20 kg, weighed on an electronic scale), according to the manufacturer's guidelines and the coefficient of variation resulted <1%. We were interested in only the positive phase of the exercise (i.e., upward/backward extension; Padulo et al., 2013a). Therefore, the participants slowly flexed downward/forward (during MP/LP) up to a starting 90° knee angle to avoid any stretch-reflex effect during the subsequent positive exercise phase (Miyaguchi and Demura, 2008).

After the warm-up, each participant was positioned for the MP with standardized flexed posture, with the 90° knee angle checked with an electrogoniometer (precision 0.01°, sampling frequency 100 Hz, MuscleLab 4020e, Bosco SystemTM, Langesund, Norway), and feedback by means of a bar just below the buttocks serving as a permanent lowest height limit (Padulo et al., 2013b). Basically, same physical measurement device handled subject's posture check and speed/surface electromyography signals measures (read below). Correspondingly to MP, during the LP, the seat was forward-blocked along its rail at different distances from the footplate according to the leg length, in order to allow the push starting knee angle to always be 90°, to match the MP starting knee angle. During both exercises, each participant was asked to push twice at maximal speed, starting with a nominal load (NL; in kg) of 20 + 17 kg of a barbell during the MP (i.e., with a corresponding *minimum* effective load = nominal load + barbel weight + participant weight), and 100+9 kg of a seat during the LP [i.e., with a corresponding *minimum* effective load = nominal load + (seat weight + participant weight)·sine(10.2°)]. Due to MP discs and LP stacks availability, load increments of 20 kg up to exhaustion were used for both exercises. The fastest repetition was chosen for further analysis. Considering the short push duration (<1 s), and to minimize any fatigue effect, a 1-min passive recovery between two same-load repetitions and a 2-min passive recovery between two subsequent loads were allowed for the participants. The participants indicated their RPE (as a value of the CR10-scale; Foster et al., 2001) after each load/exercise.

During both the MP and LP, the speed (barbell one during MP and seat one during LP) was monitored by means of a linear encoder (100 Hz, MuscleLab 4020e, Bosco SystemTM, Langesund, Norway), which recorded the upward/backward displacement over time of the barbell/seat together (synchronized) with the surface electromyography signals (sEMG) of the *vastus lateralis* (VL) and *vastus medialis* (VM). The linear encoder allowed push phase's end detection, as well. We chose to focus on two knee extensors activity coherently with our interest in only the positive phase of the exercise. Surface electromyography signals were collected using pre-amplified tri-polar leads consisting of silver-silver chloride surface electrodes (diameter—1 cm, and inter-electrode distance—1.2 cm). The EMG preparation about the skin and electrodes was performed according to the SENIAM recommendations (Hermens et al., 2000).

Raw sEMG (mV) was sampled at 1,500 Hz and band-pass filtered at 8–1,200 Hz (viz. with a Butterworth band-pass filter [3-dB low cut-off frequency 8 Hz and 3-dB high cut-off frequency 1,200 Hz]) to counteract aliasing likelihood as previously reported in the literature (Padulo et al., 2016). The filtered sEMG was then rectified and smoothed, converting it to its root mean square (sEMG_RMS_) with a 20-ms smoothing window, as previously reported in the literature (Padulo et al., 2015b). The sEMG_RMS_ signal was then re-sampled at 100 Hz using a 16-bit A/D converter and synchronized with the linear encoder used to determine the upward/backward displacement of the barbell/seat over time. Only sEMG_RMS_ recorded during dynamic trials (i.e., over the push-off phase determined by the displacement signal) was used for further analysis. Each push was analyzed by means of MuscleLab 8.0 software (Bosco SystemTM, Langesund, Norway) to obtain average speeds (m·s^−1^) and sEMG_RMS_ integral signals (mV·s).

Initially, the integrals of the sEMG_RMS_ VL and VM signals were summed to obtain a single variable, named SI, that was used to estimate the activity of both these muscles during the two exercises (i.e., MP and LP). Subsequently, different potential predictors of load were investigated, with the aim of finding the best predictor of SI during the two exercises. The potential predictors were: (1) the NLs used during the exercises, (2) the effective load (EL; in kg) taking into account the body weight (BW; in kg), and (3) and (4) the relative values of NL and EL, that were those loads normalized for the BW of each participant. For the MP the entire BW was considered as overweight for EL computation, whereas in the LP, the BW was multiplied for the sine of 10.2° (which was the inclination of the sliding axis of the leg press seat over the horizontal) in order to correctly assess the overload caused by BW during the exercise, due to the movement over an oblique axis. This part of the BW was named BW_sine10_.

Therefore, the following MP load indexes were obtained: NL_MP_ (absolute nominal load, i.e., 17 kg barbell + discs' weight); NL_MP_/BW (relative nominal load, i.e., 17 kg barbell + discs' weight, divided by BW); EL_MP_ (absolute effective load, i.e., 17 kg barbell + discs' weight + BW), and EL_MP_/BW (relative effective load, i.e., 17 kg barbell + discs' weight + BW, divided by BW). To indicate the load during the LP, the following indexes were computed: NL_LP_ (absolute nominal, that is 9 kg seat + stacks' weight); NL_LP_/BW (relative nominal load, that is 9 kg seat + stacks' weight, divided by BW); EL_LP_ (absolute effective load, that is 9 kg seat + stacks' weight + BW_sine10_), and EL_LP_/BW (relative effective load, that is 9 kg seat + stacks' weight + BW_sine10_, divided by BW).

### Statistical Analysis

Correlation analyses were performed to evaluate whether the linear model that resulted was valid to describe, for each participant, the relation between the different load indexes and SI for the MP and LP, respectively. Successively, multiple stepwise regression analyses (MSRs) were performed to evaluate the variable/s and the index/es potentially valid to predict NLs to use during the MP and LP in order to require the same muscle activity of VL and VM. Specifically, different MSRs were performed for evaluating which variable or variables were the best predictors of SI during the MP and LP trials, respectively. The variables that were significantly able to predict SI were used to calculate the conversion equation, using the respective coefficients obtained from MSRs. In particular, the analysis was carried out as follows. For the MP analysis, the SI recorded during the MP trials was used as the dependent variable, whereas NL_MP_, NL_MP_/BW, EL_MP_, EL_MP_/BW, BW, body height (BH), and age (AGE) were used as predictors. Similarly, for evaluating the variables that significantly influenced the muscle activity during the LP trials, SI was used as the dependent variable, whereas NL_LP_, NL_LP_/BW, EL_LP_, EL_LP_/BW, BW, BH, and AGE were used as predictors.

Due to the interdependence of the predictors relative to the load during the exercises, variance inflation factors (VIFs) were used to assess the multicollinearity, and when the VIFs showed that two or more predictors were collinear, these predictors were analyzed separately. Once analyzed and it was ascertained which indexes better significantly predicted SI, two successive regression analyses were performed in order to obtain two further conversion equations based on RPE scores to verify whether the coefficients of regression of SI and the coefficients of regression of RPE were similar and comparable. For these two analyses SI was used as the dependent variable, where the RPEs reported during MP and LP were considered as independent variables, and were, respectively named RPE_MP_ and RPE_LP_. The coefficients obtained by these two regression analyses were used to compute the conversion equations. Significance level was set at *P* < 0.05. Furthermore, for Pearson correlation *r* results interpretation purpose, following range was used: weak correlation when *r* was from 0 to 0.3, moderate correlation when *r* was from 0.31 to 0.7, and strong correlation when *r* was from 0.71 to 1. For all the statistical analyses, the SPSS statistical package software was used (IBM, v.23.0, Chicago, IL, USA).

## Results

Overall, 253 trials (about 8 per participant/exercise) were considered for subsequent analysis. Table 1 provides a full-comprehensive description of the two exercises in terms of used discs/stacks masses, NLs and ELs, achieved SIs and RPEs, and efforts' durations.

**Table 1:** Data relative to Smith machine squat (MP) and leg press exercise (LP) trials (Mean ± Standard Deviation).

**Smith machine squat (MP)**					
Mass of the external discs (kg)	80	100	120	140	
NL: Nominal Load (kg) (discs' weight + 17 kg barbell weight)	97	117	137	157	
EL: Effective Load (kg) (discs' weight + 17 kg barbell weight + body weight)	172.9 ± 12.5	192.9 ± 12.5	212.9 ± 12.5	232.9 ± 12.5	
SI: Integral of sEMG of *vastus lateralis* and *medialis* (mV·s)	0.712 ± 0.345	0.854 ± 0.507	0.965 ± 0.603	1.093 ± 0.723	
RPE: Rate of Perceived Exertion (score)	5.57 ± 1.70	6.54 ± 1.99	6.96 ± 1.90	7.93 ± 1.27	
Time (s)	0.72 ± 0.22	0.86 ± 0.35	0.92 ± 0.34	1.09 ± 0.41	
**Leg press exercise (LP)**					
Weight of the external stacks (kg)	60	120	180	240	300
NL: Nominal Load (kg) (stacks' weight + 9 kg seat weight)	69	129	189	249	309
EL: Effective Load (kg) (stacks' weight + 9 kg seat weight+body weight^*^)	82.2 ± 2.2	142.2 ± 2.2	202.2 ± 2.2	262.2 ± 2.2	322.2 ± 2.2
SI: Integral of sEMG of *vastus lateralis* and *medialis* (mV·s)	0.482 ± 0.221	0.626 ± 0.315	0.793 ± 0.492	0.758 ± 0.268	0.827 ± 0.389
RPE: Rate of Perceived Exertion (score)	1.15 ± 0.98	3.88 ± 1.61	6.44 ± 1.47	7.55 ± 1.19	8.12 ± 0.88
Time (s)	0.63 ± 0.1	0.66 ± 0.16	0.83 ± 0.39	0.80 ± 0.26	0.81 ± 0.36

* *In this case body weight was not entirely considered, but it was multiplied for the sine of 10° that was the inclination of the seat axis*.

Pearson's correlation analysis showed that linear correlation was a valid model to describe the relation between the load of the exercises and the relative integrals of VM and VL (*r* > 0.8 for all the participants and load indexes; Hopkins et al., 2009).

MSRs performed on MP showed that the models with NL_MP_/BW alone and with EL_MP_/BW alone can both be considered the best predictors of SI, since they had the same identical regression value (*r* = 0.632; *P* ≤ 0.01). Similarly, MSRs performed on LP showed that the model of regression with NL_LP_/BW alone, and with EL_LP_/BW alone, were the two best models to predict SI during LP (*r* = 0.603; *P* ≤ 0.01). According to these results, the two models using NL instead of EL were selected because they were easier to use in a practical way (i.e., it is easier for athletes and coaches to deal simply with discs to be fastened to Multipower barbell or with stacks to be placed into Leg press rack [i.e., NL], rather than to calculate each time EL based on BW, with Multipower, or BW and movement angle over the horizontal, with Leg press). The results of the MSRs reported in Table 2—Panel A.

**Table 2:** Multiple stepwise regression analyses for SI prediction (Panel A) and linear regression analyses on RPE (Panel B).

	**Most valid models**	**Predictors**	**SE**	**Pred. Sig**.	***r***	***r*^2^**	**Error of the estimate**	**Intercept**	**Slope**	***F*-value**	**Model Sig**.
Panel A	**MULTIPLE STEPWISE REGRESSION ANALYSIS ON PREDICTORS OF SI DURING LP**										
	1 variable: Constant, NL_LP_/BW	Constant	0.063	=0.007	0.603	0.364	0.241^a^	0.174	0.191	74.463	<0.001
		NL_LP_/BW	0.022	<0.001							
	1 variable: Constant, EL_LP_/BW	Constant	0.067	=0.038	0.603	0.364	0.241^a^	0.140	0.191	74.463	<0.001
		EL_LP_/BW	0.022	<0.001							
	**MULTIPLE STEPWISE REGRESSION ANALYSIS ON PREDICTORS OF SI DURING MP**										
	1 variable: Constant, NL_MP_/BW	Constant	0.091	=0.304	0.632	0.399	0.328^a^	0.094	0.520	53.829	<0.001
		NL_MP_/BW	0.071	<0.001							
	1 variable: Constant, EL_MP_/BW	Constant	0.159	=0.009	0.632	0.399	0.328^a^	−0.426	0.520	53.829	<0.001
		EL_MP_/BW	0.071	<0.001							
Panel B	**LINEAR REGRESSION ANALYSIS BETWEEN NL**_LP_**/BW AND RPE DURING LP**										
	Constant, NL_LP_/BW	Constant	0.367	<0.001	0.726	0.527	1.483^b^	1.641	1.646	165.946	<0.001
		NL_LP/_BW	0.128	<0.001							
	Constant, EL_LP_/BW	Constant	0.388	=0.001	0.726	0.527	1.483^b^	1.349	1.646	165.946	<0.001
		EL_LP/_BW	0.128	<0.001							
	**LINEAR REGRESSION ANALYSIS BETWEEN NL**_MP_**/BW AND RPE DURING MP**										
	Constant, NL_MP_/BW	Constant	0.368	=0.762	0.852	0.727	1.394^b^	−0.112	3.998	220.568	<0.001
		NL_MP_/BW	0.269	<0.001							
	Constant, EL_MP_/BW	Constant	0.623	<0.001	0.852	0.727	1.394^b^	−4.110	3.998	220.568	<0.001
		EL_MP_/BW	0.269	<0.001							

SE, Standard Error of each predictor; Pred. Sig., significance of each predictor; Model Sig., significance of the model.

a This error of estimate is expressed as SI (mV·s);

b *This error of estimate is expressed as RPE (score)*.

According to the constants and coefficients of this MSRs, the following conversion equations based on SI were obtained:

$$\begin{array}{l} \text{N}{\text{L}}_{\text{MP}}/\text{BW}=\left(0.36\times \text{N}{\text{L}}_{\text{LP}}/\text{BW}\right)+0.33\text{ or}\\ \text{ N}{\text{L}}_{\text{LP}}/\text{BW}=\left(2.73\times \text{N}{\text{L}}_{\text{MP}}/\text{BW}\right)-0.90 \end{array}$$

which imply:

$$\begin{array}{l} \text{N}{\text{L}}_{\text{MP}}=0.36\times \text{N}{\text{L}}_{\text{LP}}+0.33\times \text{BW or}\\ \text{ N}{\text{L}}_{\text{LP}}=2.73\times \text{N}{\text{L}}_{\text{MP}}-0.90\times \text{BW} \end{array}$$

The two variables of the models obtained by means of MSRs were successively used for the NL_MP_/BW vs. RPE_MP_, and NL_LP_/BW vs. RPE_LP_ regression analyses. These regression analyses showed that NL_MP_/BW and NL_LP_/BW are strongly correlated with RPE (with *r* = 0.852 and *r* = 0.726, respectively). Details of these analyses are reported in Table 2—Panel B. In this case, the following conversion equations, based on RPE, were obtained:

$$\begin{array}{l} \text{N}{\text{L}}_{\text{MP}}/\text{BW}=\left(0.41\times \text{N}{\text{L}}_{\text{LP}}/\text{BW}\right)+0.41\text{ or}\\ \text{ N}{\text{L}}_{\text{LP}}/\text{BW}=\left(2.42\times \text{N}{\text{L}}_{\text{MP}}/\text{BW}\right)-1.00 \end{array}$$

which imply:

$$\begin{array}{l} \text{N}{\text{L}}_{\text{MP}}=0.41\times \text{N}{\text{L}}_{\text{LP}}+0.41\times \text{BW or}\\ \text{ N}{\text{L}}_{\text{LP}}=2.42\times \text{N}{\text{L}}_{\text{MP}}-\text{BW} \end{array}$$

## Discussion

The main and novel contribution of the present study is the practical and immediate method of calculating the respective NLs between the MP and LP, thus achieving the same muscle activity of VL and VM. However, these equations showed a not-negligible standard error in assessing SI, and the values computed with these equations can have a moderate margin of error due to the elevated error of the estimate of the predictive models. The elevated error of the estimate is probably due to the high inter-individual differences of the participants, as suggested by the strong correlation obtained from the Pearson's analysis (Table 2). During both exercises, the linear increment of the NL demanded a linear increment of the muscle activity, but the amount of increment of muscle activity, as well as the resting muscle activity, are strongly subject-specific, and for this reason it is difficult to find a single equation that can predict the muscle activity of VL and VM. In fact, the muscle activity is not correlated with BW, but probably depends on a number of factors that are not fully measurable, such as level of expertise in this specific kind of training, intramuscular coordination, ability to recruit other muscles to support and stabilize the muscle activity of the VL and VM during the MP and LP, and anthropometric differences among people that can greatly influence the complex biomechanics of these two kinds of exercise (Escamilla et al., 2001). Another factor to consider is that muscle sEMG activity differed strongly between different body weight unloading conditions (such as during our LP), as showed by previous studies (Dietz and Colombo, 1998; Van Hedel et al., 2006).

In contrast, the equation obtained for RPE showed high regression coefficients. Probably, RPE is more related to the overall level of exertion demanded by the two exercises compared with SI. This finding is in agreement with previous literature indicating that RPE is a reliable method to quantify various intensities of resistance training as well as muscle activity (Lagally et al., 2002, 2004; Day et al., 2004). RPE might better reflect recruitment of other muscles supporting and stabilizing the muscle activity of the VL and VM during the MP and LP. This finding is in agreement with a previous study of Escamilla et al. (2001) that found greater muscle activity and knee force in the squat exercise compared with LP.

A further confirmation supporting these important differences between the two exercises comes from some further examination of the equations calculated for RPE and SI. In fact, a discrepancy exists between the NLs to be used during the MP and LP in order to require the same SI or achieve the same RPE. To better understand this point, it is useful to provide an example: if a typical 70-kg subject uses a 100-kg NL during the MP, the conversion equations would suggest that he/she should use a 210-kg NL during the LP in order to match the same SI (i.e., LP/MP NL *ratio* 2.1) but a 172-kg NL to achieve the same RPE (i.e., LP/MP NL *ratio* 1.7). In another case, if the same subject would use a 150-kg NL during the MP, the conversion equations would suggest that he/she should use a 347-kg NL during LP in order to match the a same SI (i.e., LP/MP NL *ratio* 2.3) but a 293-kg NL to achieve the same RPE (i.e., LP/MP NL *ratio* 2.0). To summarize, during the LP, in order to demand a SI similar to that of MP, a NL higher than that necessary to achieve an equal RPE is required. Given the low number of muscles investigated in the present study, a speculative explanation is that the MP engages a higher number of muscles (e.g., trunk stabilizers; Graham et al., 1993), and consequently this exercise always needs a NL lower than the LP's one in order to be considered as equally strenuous in terms of RPE, despite the fact that the MP's NL does not need to be proportionally lower than the LP's in order to achieve the same SI. In other words (and limited to RPE), VL and VM may not be fully representative of the effort sustained during the two investigated exercises. For example, the contribution of a large hip extensor muscle is not considered in the same SI-based equation, whereas it accounts for the corresponding RPE-based one.

As a data processing limitation of the current study, we acknowledge sEMG_RMS_ signal 100 Hz re-sampling might have biased results. Proper processing was simply applying a 15-ms smoothing window to homogenize EMG and linear encoder measurements. As another study limitation, we have to acknowledge that since LP presents a high variability (in terms of movement angle over the horizontal), this makes it difficult to apply the findings to all the different kinds of LP machines that are in use (i.e., horizontal, vertical, and differently inclined leg presses). In fact, NLs used during a LP demand a different muscular workload that is strictly dependent on the LP machine type used to perform the exercise (e.g., with athlete's whole body movement, only the athlete's legs movement, the load moving over an inclined plane, stacks moving vertically). However, the results are still useful in providing an immediate, cost-effective, and practical way to of adjusting the two training modes' nominal loads during everyday practice. For MP, whole BW was considered as overweight for EL computation. We acknowledge that is in contrast with some author finding (Winter, 2009), i.e., that lower limb mass, accounting for a small overall body weight fraction, should not be considered as overweight regarding counter-gravity movements. Yet, we preferred to keep equation components as simple as possible to provide athletes and coaches with easy-to-use practical indications at the price of some accuracy decrease. Further limitation of the study is that—coherently with our interest in only the positive phase of the exercise—we focused on two knee extensors activity, whereas choosing an antagonist or other joints' muscles would have provided information more valuable within a motor control perspective. We could investigate also “accessory” muscles with supporting/stabilizing function of main muscles object of study. Future studies may be featured by different muscles choice. We are aware sEMG vs. force relationship is nonlinear in many cases in accordance with Alkner et al. (2000). However, our preliminary investigation showed a linear model was able to significantly describe this relationship in most of our participants. We are aware this is not an absolute condition and consequently a nonlinear sEMG vs. force relationship could result to be a limitation in the use of our equation. About our obtained conversion equations, we believe they represent a starting result, which might be improved by further investigations. Final study limitation regards checking for equation-provided NLs at one-repetition *maximum* intensity. We mean that once we found the two same muscle activation and same rating of perceived exertion-driven equations (i.e., after both data collection and analysis), we omitted testing on our subjects—due to their ceased availability—their validity at one-repetition *maximum* intensity. We are confident equations work at one-repetition *maximum* intensity as well, but we cannot state that. Therefore, we believe that can be matter for further studies in order to provide the reader with that practical indication as well. Future investigations might focus on dominant vs. non-dominant lower limb answer to both exercises, to further inform about two machined' use.

## Conclusions

Further research is needed to improve the same muscle activation equation accuracy, due to its high sensitivity to the participants' inter-individual differences. Although the same rating of perceived exertion equation already provides good accuracy, the two equations represent a preliminary attempt to provide athletes and their coaches with a new tool for assisting in decision making concerning rehabilitation program planning, and to provide input for training strategies.

## Author Contributions

All authors listed have made a substantial, direct and intellectual contribution to the work, and approved it for publication.

### Conflict of Interest Statement

The authors declare that the research was conducted in the absence of any commercial or financial relationships that could be construed as a potential conflict of interest.

## Abbreviations

AGE: age
BH: body height
BW: body weight
BW_sine10_: body weight's vertical part featuring leg press exercise due to its movement over an oblique axis
CKC: closed kinetic-chain exercise
EL: effective load
EL_LP_: effective load during leg press exercise
EL_MP_: effective load during squat exercise performed at the Smith machine Multipower
EMG: electromyography activity
LP: leg press exercise
MP: squat exercise performed at the Smith machine Multipower
MSR: multiple stepwise regression analysis
NL: nominal load
NL_LP_: nominal load during leg press exercise
NL_MP_: nominal load during squat exercise performed at the Smith machine Multipower
OKC: open kinetic chain exercise
RPE: rating of perceived exertion
sEMG_RMS_: filtered and rectified sEMG converted to its root mean square
SI: sum of integrals of the sEMG_RMS_s of *vastus lateralis* and *vastus medialis*
VIF: variance inflation factor
VL: *vastus lateralis*
VM: *vastus medialis*.
