# Corrigendum: Leg Press vs. Smith Machine: Quadriceps Activation and Overall Perceived Effort Profiles

**DOI:** 10.3389/fphys.2018.01856

**Published:** 2018-12-21

**Authors:** Gian Mario Migliaccio, Antonio Dello Iacono, Luca Paolo Ardigò, Pierre Samozino, Enzo Iuliano, Zoran Grgantov, Johnny Padulo

**Affiliations:** ^1^Sport Science Lab, Cagliari, Italy; ^2^Wingate Institute, The Academic College at Wingate, Netanya, Israel; ^3^Institute of Clinical Exercise and Health Science, School of Health and Life Sciences, University of the West of Scotland, Hamilton, United Kingdom; ^4^Department of Neurosciences, School of Exercise and Sport Science, Biomedicine and Movement Sciences, University of Verona, Verona, Italy; ^5^Laboratoire Interuniversitaire de Biologie de la Motricité, EA 7424, Université Savoie Mont Blanc, Chambéry, France; ^6^Faculty of Psychology, eCampus University, Novedrate, Italy; ^7^Faculty of Kinesiology, University of Split, Split, Croatia

**Keywords:** muscle strength, strength exercise, workload, resistance training, exercise intensity

In the published article, there was an error in the affiliation for Zoran Grgantov. Instead of affiliation 6, “Faculty of Psychology, eCampus University, Novedrate, Italy,” the correct affiliation for Zoran Grgantov should be affiliation 7, “Faculty of Kinesiology, University of Split, Split, Croatia”.

The authors apologize for this error and state that this does not change the scientific conclusions of the article in any way. The original article has been updated.

